# Relationships Between Phenotype and Function of Blood CD4^+^ T-Cells and Ascending Thoracic Aortic Aneurysm: an Experimental Study

**DOI:** 10.21470/1678-9741-2018-0310

**Published:** 2019

**Authors:** Silverio Sbrana, Kaushal Kishore Tiwari, Stefano Bevilacqua, Paola Giungato, Enkel Kallushi, Marco Solinas, Anna Maria Mazzone

**Affiliations:** 1 Flow Cytometry Laboratory, CNR Institute of Clinical Physiology, Massa, Italy.; 2 Cardiac Surgery Department "G. Pasquinucci" Heart Hospital, "G. Monasterio" Foundation, Massa, Italy.; 3 Institute of Life Sciences, Scuola Superiore S. Anna, Pisa, Italy.; 4 Department of Cardiothoracic and Vascular Surgery, College of Medical Sciences, Teaching Hospital, Bharatpur, Chitwan, Nepal.; 5 Cellular Biology Laboratory, CNR Institute of Biomedical Technologies, Pisa, Italy.; 6 Cardiology Department "G. Pasquinucci" Heart Hospital, "G. Monasterio" Foundation, Massa, Italy.

**Keywords:** Aortic Aneurysm, Thoracic, CD4-Positive T-Lymphocytes, Flow Cytometry

## Abstract

**Introduction:**

Non-familial ascending thoracic aorta dilation and aneurysms (TAAs) are silent diseases in elderly patients. Histopathology revealed that functionally polarized infiltrating CD4^+^ T-cells play a key role in aortic wall weakening.

**Objective:**

To evaluate the possible associations between phenotype and cytokine production of circulating CD4^+^ T-lymphocytes and the presence of TAA in patients with aortic valve disease (AVD).

**Methods:**

We studied blood samples from 10 patients with TAA and 10 patients with AVD. Flow cytometry was used to quantify: a) CD4^+^ T-lymphocytes surface expression of CD25, CD28, and chemokine receptors (CCR5, CXCR3, CX3CR1); b) fractions of *in vitro* stimulated CD4^+^ T-cells producing cytokines (interferon gamma [IFN-γ], interleukin [IL]-17A, IL-21, IL-10); c) CD4^+^CD25^high^FoxP3^+^ regulatory T-cells (Treg) fraction. Enzyme-linked immunosorbent assays (ELISA) were performed for cytokines (IFN-γ, IL-6, IL-10, IL-17A, IL-23, transforming growth factor beta [TGF-β]) and chemokines (RANTES, CX3CL1).

**Results:**

The total CD4^+^CD28^±^CD4^+^/CX3CR1^+^ T-cells fraction was higher (*P*=0.0323) in AVD (20.452±4.673) than in TAA patients (8.633±2.030). The frequency ratio of CD4^+^ T-lymphocytes producing IFN-γ *vs*. IL-17A+IL-21 cytokine-producing CD4+ T-cells was higher (*P*=0.0239) in AVD (2.102±0.272) than in TAA (1.365±0.123) patients. The sum of CD4^+^CD28^±^CD4^+^/CX3CR1^+^ T-cells correlated positively with values of the previous cytokine ratio (*P*=0.0002, R=0.732). The ratio of CD4^+^CD28^±^CD4^+^/CX3CR1^+^ T-cells *vs*. Treg was higher (*P*=0.0008) in AVD (20.859±3.393) than in TAA (6.367±1.277) patients.

**Conclusion:**

Our results show that the presence of TAA in subjects with AVD is associated with imbalance between phenotypic and cytokine-producing subsets of circulating CD4+ T-lymphocytes, prevalently oriented towards a pro-fibrotic and IFN-γ counteracting effect to functional polarization.

**Table:** 

Abbreviations, acronyms & symbols				
ACE	= Angiotensin-converting enzyme		K_3_-EDTA	= Tripotassium ethylenediaminetetraacetic acid
ANOVA	= Analysis of variance		MF	= Myofibroblasts
APC	= Antigen-presenting cells		PE	= Phycoerythrin
AVD	= Aortic valve disease		PMA	= Phorbol myristate acetate
AVR	= Aortic valve replacement		RPMI	= Roswell Park Memorial Institute
Cy5	= Cyanine 5		SEM	= Standard errors of the mean
ECM	= Extracellular matrix		SSC	= Side scatter
ELISA	= Enzyme-linked immunosorbent assay		TAA	= Thoracic aortic aneurysm
FITC	= Fluorescein isothiocyanate		TAS	= Thoracic aortic surgery for aneurysm
FoxP3	= Forkhead-box-P3		TGF-β	= Transforming growth factor beta
IFN-γ	= Interferon gamma		Treg	= Regulatory T-cells
IL	= Interleukin		WBC	= White blood cells

## INTRODUCTION

The non-familial ascending thoracic aortic dilatations and thoracic aortic aneurysms (TAA) are frequent in individuals older than 65 years of age (approximately 6-9%), with a risk of rupture or dissection ranging from 2 to 3.5 cases per 100,000 patients/year^[1]^.

The progression from aortic dilatation to aneurysm is a multifactorial process partially undiscovered. Besides other well-known mechanisms^[2]^, the role of chronic immune-mediated inflammations in defining biomechanical properties of the aortic wall is still to be determined. In particular, the study of cellular and molecular mechanisms leading to aortic fibrosis, considered the histopathological marker of an altered vascular remodeling process^[3]^, might be an important target in understanding the individual's susceptibility to non-syndromic ascending thoracic aortic dilatation and TAA formation. Recent findings in humans confirmed the key role of myofibroblasts (MF) in the extracellular matrix (ECM) proteolysis and deposition (fibrosis)^[4]^. MF activity is modulated by a wide array of pro- and anti-fibrotic cytokines and growth factors released by mononuclear immune cells infiltrating the inflamed tissue^[5]^. Especially, the cytokines produced by CD4^+^ T-lymphocytes play a causative role in the initiation and progression of fibrosis associated with pathological conditions such as systemic sclerosis, atherosclerosis, and the use of silicone mammary implants^[6]^. Given the crucial role of distinct CD4^+^ T-lymphocyte subsets in normal immune-regulation^[7]^, the evaluation of their dynamic functional balance in terms of cell frequency ratio, either expressing a particular surface phenotype or producing selective signature cytokines, is an important tool to investigate whether a clinical pathological condition and its outcome are associated with a particular T-helper functional perturbation^[8,9]^.

To our knowledge, so far, no studies have related phenotype and function of peripheral blood CD4^+^ T-lymphocytes with the presence of TAA. Therefore, we have evaluated the presence of possible relationships between this pathological condition and: a) the expression of chemokine receptors and activation markers (CCR5, CXCR3, CX3CR1, CD25) on total blood CD4^+^ T-cell and on the pro-inflammatory/cytotoxic subset CD4^+^CD28^-^, known to be involved in vascular inflammation^[10]^; b) the cytokine production (interferon gamma [IFN-g, interleukin [IL]-17A, IL-21, IL-10) by *in vitro* stimulated CD4^+^ T-cells. Moreover, given their both beneficial and harmful roles in several clinical settings, including vascular injury^[11]^, we quantified the circulating fraction of the CD4^+^CD25^high^FoxP3^+^ naturally occurring regulatory T-cells (Treg). On this basis, newly established phenotypic and functional blood CD4^+^ T-lymphocyte ratios, that overcome the traditional Th1/Th2 paradigm^[12]^, have been calculated and related to the presence of an aortic aneurysm. We also measured the circulating levels of several cytokines (IFN-γ, IL-6, IL-10, IL-17A, IL-23, transforming growth factor beta [TGF-β]) and chemokines (RANTES, CX3CL1) known to influence CD4^+^ T-cells function and migration^[13-15]^.

## METHODS

### Patients and Blood Samples

We have enrolled 20 patients undergoing surgery for aortic valve disease (AVD). TAA group (n=10) included patients undergoing aortic valve surgery with TAA surgery and AVD group (n=10) included patients undergoing surgery only for AVD, like stenosis or regurgitation, or both. Both groups underwent surgery by aortotomy. All patients had an indication for aortic valve surgery, according to current guidelines. TAA surgery was performed, if recommended by current guidelines. Exclusion criteria were the presence of genetic disorders, autoimmune and chronic inflammatory diseases, cancer, or hematological diseases. We enrolled patients with normal, ectasic or entirely aneurysmatic ascending aorta. Comorbidities, risk factors, and medical therapies were recorded, with particular attention to statins and/or angiotensin-converting enzyme (ACE) inhibitors, known to exert also immunomodulating effects^[16-18]^. Mean and peak aortic gradients were measured by Doppler echocardiography and used to define the grade of stenosis. Aortic valve lesions were classified as predominant stenosis (mean transvalvular gradient ≥40 mmHg and grade of regurgitation <3^+^/4^+^), predominant regurgitation (grade of regurgitation 4^+^/4^+^ and mean gradient <40 mmHg), and steno-regurgitation, in other cases.

Venous blood was collected at the admission in Vacutainer tubes containing tripotassium ethylenediaminetetraacetic acid (K_3_-EDTA) or sodium-heparin as anticoagulants. Plasma was obtained after blood centrifugation and immediately frozen at -80ºC. The protocol was approved by the local ethics committee and informed consent was obtained from each patient.

### Data Acquisition and Analysis

Leukocyte count was carried out with a routine analyzer. Flow cytometry was performed with a FACS scan instrument equipped with a CellQuest software (Becton Dickinson). Quantification of lymphocyte surface markers expression and intracellular cytokines content was based on measurement of fraction (percentage) of positive events. Enzyme-linked immunosorbent assays (ELISA) were performed using the ASYS HITECH Microplate Reader (Eugendorf).

### Surface Staining

**Fig. 1:**
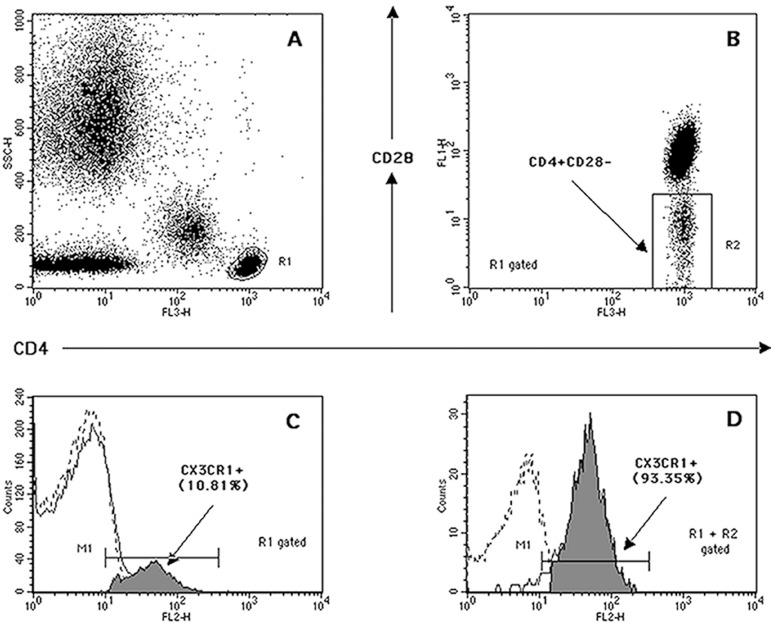
Identification of total CD4^+^ and CD4^+^CD28- T-lymphocyte subsets. Top: Selection of total CD4^+^ T-lymphocytes (R1) based on side scatter (SSC) properties and FL3 (CD4) bright fluorescence (A). R1-derived dot-plot of FL1 (CD28) versus FL3 (CD4) used to identify and quantify (as percentage) the CD4^+^CD28^-^ T-lymphocyte fraction (R2) (B). Bottom: Example of quantification of CX3CR1 expression. The overlay of the FL2 fluorescence histograms is used to quantify the CX3CR1 positive cells (as percentage, events in M1, filled histograms) on the selected total CD4^+^ (R1 gated) (C) and CD4^+^CD28- (R1 + R2 gated) (D) T-cell subsets, when compared with isotype controls (dotted histograms).

Blood samples were processed as previously described for monocyte analysis^[19]^. In brief, K_3-E_DTA samples were treated with a red cell lysing solution to isolate leukocytes, and then stained with the following combinations of phycoerythrin (PE)/cyanine 5 (Cy5) [PC5]-, fluorescein isothiocyanate (FITC)-, and (PE)-conjugated monoclonal antibodies: CD4/CD28/CD25; CD4/CD28/CCR5; CD4/CD28/CXCR3; CD4/CD28/CX3CR1. Isotype controls were performed. The antibodies used were from Becton Dickinson, Pharmingen, Immunotech, R&D Systems, and MBL International Corporation. The acquisition was stopped after 30,000 CD4^+^ T-lymphocytes were collected for each sample (Figure 1).

### Intracellular Cytokines

**Fig. 2:**
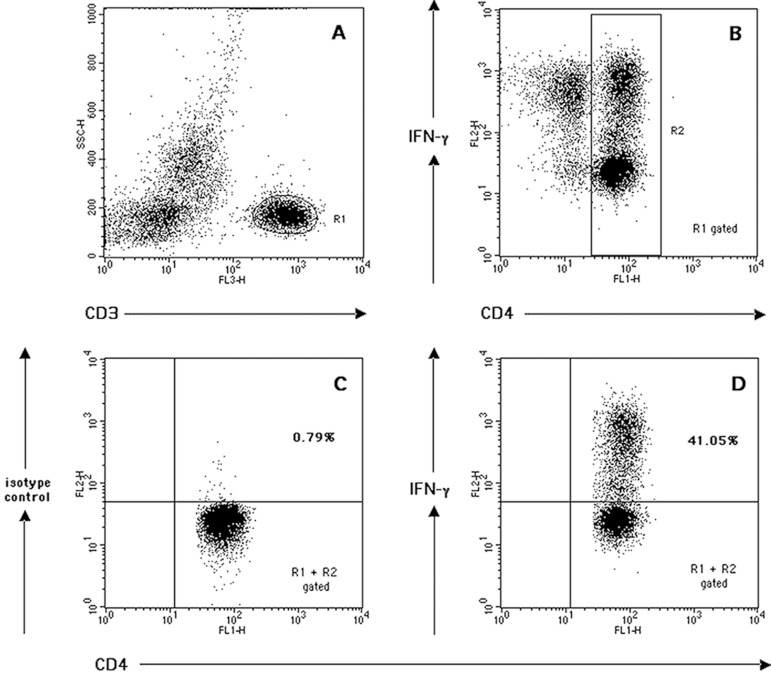
Analysis of intracellular interferon gamma (IFN-y) production by CD4^+^ T-lymphocytes in activated whole blood samples, as example of cytokine-producing cells quantification by dot-plot quadrant statistics. Top: Selection of CD3^+^ T-lymphocytes (R1) based on side scatter (SSC) properties and FL3 (CD3) bright fluorescence (A). R1-derived dot-plot of FL2 (IFN-γ) vs. FL1 (CD4) used to select the CD4^+^ T-lymphocyte fraction (R2) (B). Bottom: Quantification (as percentage) of CD4^+^ T-cells producing IFN-γ (upper right quadrant, R1 + R2 gated) (D), when compared with isotype control (upper right quadrant, R1 + R2 gated) (C).

A whole blood staining procedure for intracellular cytokines detection was carried out as described^[20]^. In brief, heparinized samples were diluted 1:1 with RPMI (acronym for Roswell Park Memorial Institute) medium complete medium and incubated at a density of 2x10^6^ leukocytes/ml for 5 hours, at 37ºC. Phorbol myristate acetate (PMA) (Sigma Chemical Co.) (50 ng/ml) and ionomycin (1 mg/ml) were used as *in vitro* activators, in the presence of brefeldin A (eBioscience Inc.) (3 mg/ml final) as secretion blocking agent. Samples were then recovered, treated with a red cell lysing solution, and the isolated white blood cells (WBC) were stained with a combination of PC5- and FITC-conjugated monoclonal antibodies against the markers CD3 and CD4, respectively. Then, the cells were fixed with formaldehyde (2%) and permeabilized with saponin (0.5%). Cytokine staining was performed with PE-labeled monoclonal antibodies against IL-17A, IL-21, IFN-γ and IL-10. Isotype controls were performed. The acquisition was stopped after 30,000 CD3^+^ T-lymphocytes were collected for each sample (Figure 2).

### CD4^+^CD25^high^FoxP3^+^ Regulatory T-cells

**Fig. 3:**
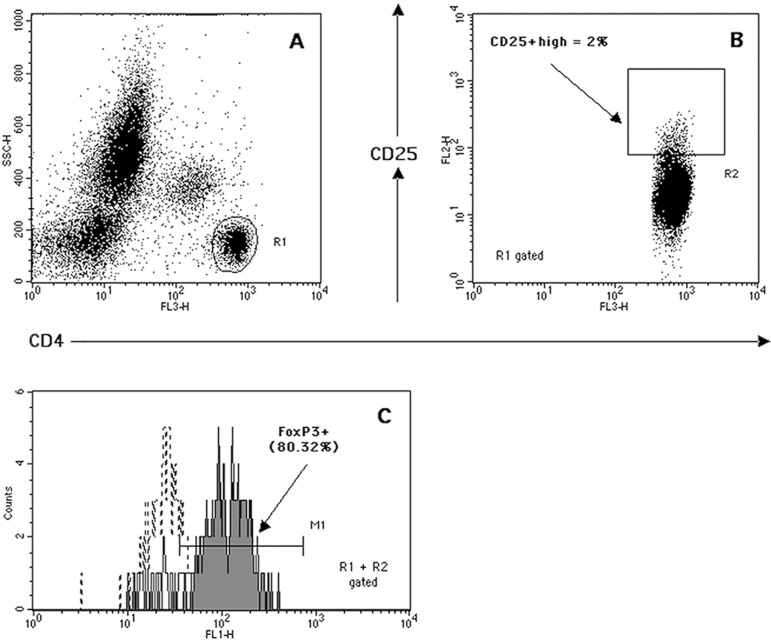
Gating sequence used to quantify the frequency of regulatory T-cells (Treg). Top: Identification of CD4^+^ T-lymphocytes (R1) based on side scatter (SSC) properties and FL3 (CD4) bright fluorescence (A). R1-derived dot-plot of FL2 (CD25) vs. FL3 (CD4) used to select the CD25^high^ positive events at the top 2% of CD4^+^CD25^+^ T-cells (R2) (B). Bottom: The overlay of Alexa Fluor-488 FL1 fluorescence histograms is used to quantify the percentage of FoxP3^+^ cells (events in M1, filled histogram) (C), when compared with isotype control (dotted histogram). By multiplying the above 2 percentages and dividing them by 100, the CD4^+^CD25^high^FoxP3^+^ (Treg) cell fraction is calculated.

Blood samples were processed as published^[21]^, with minor modifications. In brief, K_3_-EDTA samples were lysed as described before and the isolated WBC stained with PC5- and PE-conjugated monoclonal antibodies against the markers CD4 and CD25, respectively. Then, cells were fixed, permeabilized, and stained with an Alexa Fluor 488-conjugated anti-human forkhead-box-P3 (FoxP3) monoclonal antibody (eBioscience Inc.). Isotype controls were performed. Treg cells fraction was quantified into the 2% of CD4^+^CD25^high^ double positive events, as reported^[22]^. The acquisition was stopped after 30,000 CD4^+^ T-lymphocytes were collected for each sample (Figure 3).

### ELISA

Plasma levels of IFN-γ, IL-6, IL-10, IL-17A, IL-23, TGF-β, RANTES, and CX3CL1 were quantified by ELISA kits (R&D Systems) according to the manufacturer's protocol. Minimum detectable concentrations were 15.6 pg/ml (IFN-γ, 0.156 pg/ml (IL-6), 0.78 pg/ml (IL-10), 31.25 pg/ml (IL-17A), 39.0 pg/ml (IL-23), 0.031 ng/ml (TGF-β, 0.031 ng/ml (RANTES), and 0.156 ng/ml (CX3CL1).

### Statistical Analysis

The calculation of mean values and standard errors of the mean (± SEM), as well as the determination of linear correlations among continuous variables, was carried out with the StatView 5.0 software. The existence of statistically significant differences between groups was explored by analysis of variance (ANOVA) Student's t-test for unpaired data. Chi-squares statistical analysis was used for comparison between nominal variables. *P*<0.05 was considered statistically significant.

## RESULTS

### General Findings

Mean aortic diameters were 35.50 ± 1.65 mm (range 27-43 mm) and 48.90 ± 1.18 mm (range 45-56 mm) in AVD and TAA groups, respectively (*P*<0.0001). At the operation, 8 patients had an isolated ascending aorta replacement and 2 underwent combined aortic root surgery. Clinical and surgical characteristics of patients are reported in Table 1.

**Table 1:** Patients' clinical characteristics (n=20).

Variables	Values (mean ± SEM) or number (%)
Age (years)	67.9±1.33
Sex (male)	12 (60%)
Smoker	11 (55%)
Obesity	7 (35%)
Diabetes	2 (10%)
Hypertension	13 (65%)
Statin therapy	12 (60%)
Angiotensin-converting enzyme inhibitors therapy	6 (30%)
Predominant aortic valve pathology	
Bicuspid	6 (30%)
Regurgitation	8 (40%)
Stenosis	10 (50%)
Steno-regurgitation	2 (10%)
Mean gradient* (mmHg)	51.50±2.70
Type of surgery	
Isolated AVR	10 (50%)
AVR + TAS	10 (50%)

* Calculated in aortic stenosis and steno-regurgitation.

AVR=aortic valve replacement; SEM=standard error of the mean; TAS=thoracic aortic surgery for aneurysm

The mean frequency (± SEM) of CD4^+^ T-lymphocytes was 46.39±1.99; their absolute number (number of cells/ml) was 953.34±61.85, accordingly with published CD4^+^ T-cell reference values during aging^[23]^. The mean percentage (± SEM) of CD4^+^ T-cells belonging to the pro-inflammatory/cytotoxic subset CD4^+^CD28^-^ was 5.56±1.33; this value, if referred to the age's range of our patients, is also in accord with literature data^[24]^. Cumulative analysis of surface markers expression on total CD4^+^ and CD4^+^CD28^-^ T-cell subsets are reported in Table 2.

**Table 2:** Surface marker expression on blood total CD4^+^and CD4^+^CD28- T-cell subsets (percentage of positivity, mean ±SEM) (20 patients).

	CD25	CCR5	CXCR3	CX3CR1
Total CD4^+^	42.21±1.64	14.51±0.82	44.65±2.54	8.98±1.54
CD4^+^CD28^-^	3.98±2.64	28.65±2.42	71.75±3.70	91.77±1.45

SEM=standard error of the mean

The measurement of plasma cytokines/chemokines is reported in Table 3.

**Table 3:** Blood levels of cytokines and chemokines (mean ± SEM) (20 patients).

Variables	Mean ± SEM
IFN-γ (pg/ml)	10.36±3.25
IL-6 (pg/ml)	2.65±0.50
IL-17A (pg/ml)	13.51±2.37
IL-23 (pg/ml)	20.76±3.90
IL-10 (pg/ml)	0.55±0.07
RANTES (ng/ml)	17.35±1.52
TGF-β (ng/ml)	6.59±0.58
CX3CL1 (ng/ml)	0.47±0.02

IFN-γ=interferon gamma; IL=interleukin; SEM=standard error of the mean; TGF β=transforming growth factor beta

No relationship has been found between the plasma levels of cytokines/chemokines and CD4^+^ T-cells phenotypes, intracellular cytokines levels, and presence of TAA, respectively. Cumulative quantification of intracellular cytokines is reported in Table 4; also, these data are in accordance with published T-cell cytokines levels in normal aging^[25]^.

**Table 4:** Intracellular cytokine production by "*in vitro*" stimulated CD4^+^ T-lymphocytes in whole blood samples (percentage of positivity, mean ± SEM) (20 patients).

	IFN-γ	IL-10	IL-17A	IL-21
CD4^+^ T-cells	24.84±3.13	2.70±0.38	1.86±0.22	14.02±1.46

IFN-γ=interferon gamma; IL=interleukin; SEM=standard error of the mean

### Relationships among CD4^+^ T-cells Phenotypic Subsets

The accumulation of CD28^-^ T-cells in aging is driven by a repeated cellular activation, leading also to a modulation of CD4^+^ T-cells chemokine receptors expression^[24,26]^. In our patients, a tightly positive correlation between the circulating fraction of CD4^+^CD28^-^ T-cells and the frequency of CD4^+^ T-lymphocytes carrying the fractalkine receptor CX3CR1 (CD4^+^/CX3CR1^+^) (*P*<0.0001, R=0.934) has been observed.

### Relationships among CD4^+^ T-cells Phenotype/Function and TAA

**Fig. 4:**
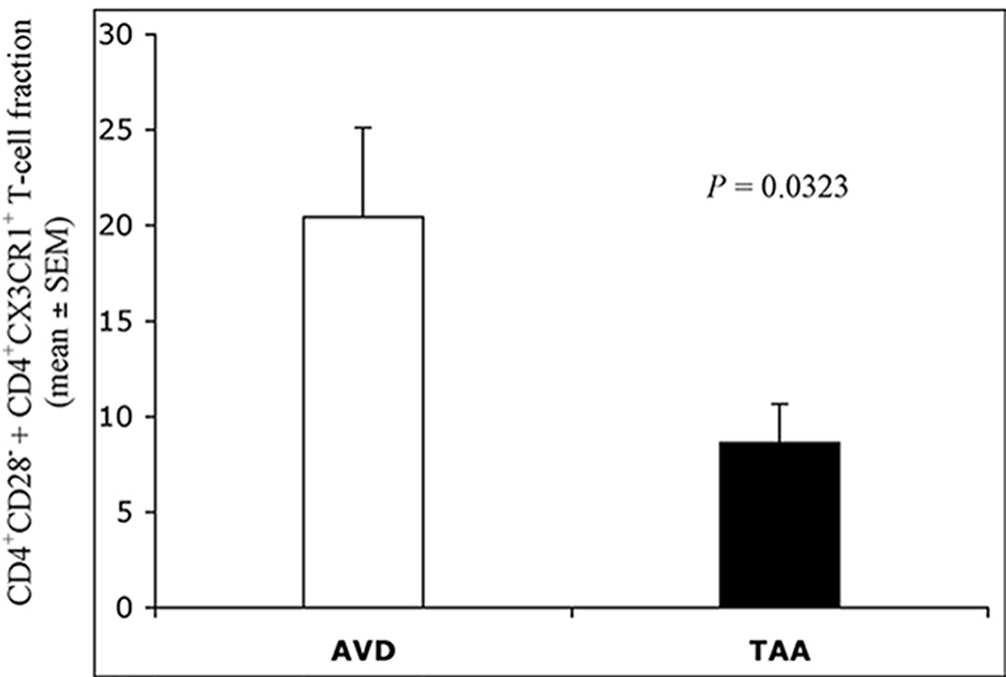
Total fraction (percentage) of circulating CD4^+^CD28^±^CD4^+^/CX3CR1^+^ T-cells in AVD (white bar) and TAA (black bar) patients. Statistical comparison between percentages (mean ± SEM) was performed with ANOVA Student's t-test for unpaired data. Statistically significant differences (P<0.05) were detected. The functional balance (ratio) between the blood frequency of CD4^+^ T-lymphocytes producing IFN-γ vs. the total fraction of IL-17A+IL-21 cytokine-producing CD4^+^ T-cells was significantly lower in the TAA group (Figure 5A). ANOVA=analysis of variance; AVD=aortic valve disease; IFN-γ=interferon gamma; IL=interleukin; SEM=standard error of the mean; TAA=thoracic aortic aneurysm

The sum of blood CD4^+^CD28±CD4^+^/CX3CR1^+^ T-cells fractions was significantly lower in the TAA group than in the AVD group (Figure 4).

**Fig. 5:**
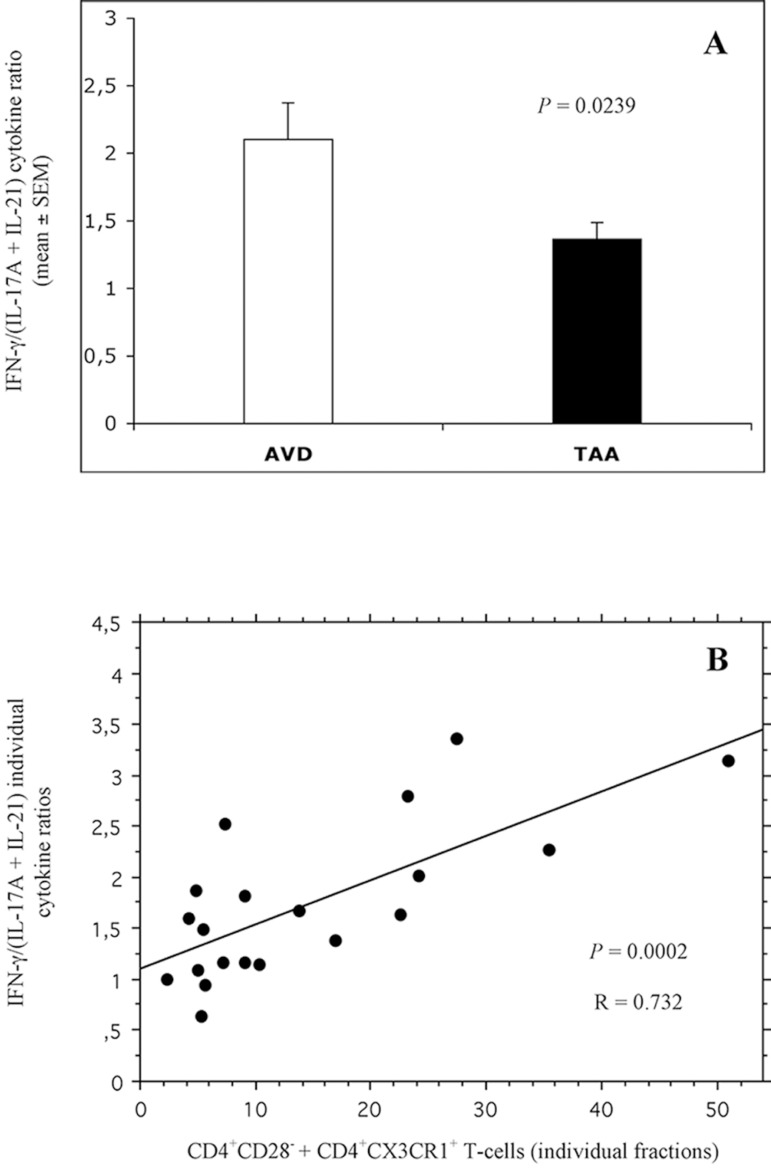
(A) Ratio between the blood frequency of CD4^+^ T-lymphocytes producing IFN-γ vs. the total fraction of IL-17A+IL-21 cytokine-producing CD4^+^ T-cells in AVD (white bar) and TAA (black bar) patients. Statistical comparison between ratios (mean ± SEM) was performed with ANOVA Student's t-test for unpaired data. Statistically significant differences (P<0.05) were detected. (B) Linear regression between the individual fractions (percentages) of circulating CD4^+^CD28^±^CD4^+^/CX3CR1^+^ T-cells (x axis) and the corresponding IFN-γ/(IL-17A+IL-21) cytokine ratios (y axis) in all patients. ANOVA=analysis of variance; AVD=aortic valve disease; IFN-γ=interferon gamma; IL=interleukin; SEM=standard error of the mean; TAA=thoracic aortic aneurysm

For each patient, the sum of CD4^+^CD28^±^CD4^+^/CX3CR1^+^ T-cell fractions correlated positively with the corresponding values of the above-mentioned cytokine ratio (Figure 5).

**Fig. 6:**
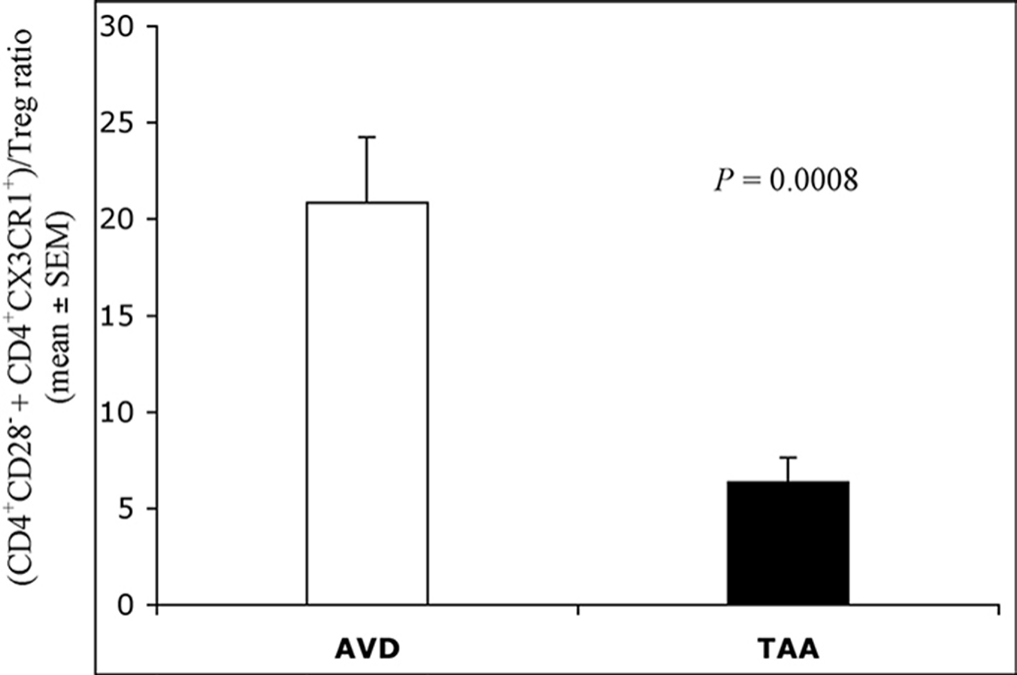
Frequency ratio of blood CD4^+^CD28^±^CD4^+^/CX3CR1^+^ T-cells vs. Treg in AVD (white bar) and TAA (black bar) patients. Statistical comparison between ratios (mean ± SEM) was performed with ANOVA Student's t-test for unpaired data. Statistically significant differences (P<0.05) were detected. ANOVA=analysis of variance; AVD=aortic valve disease; SEM=standard error of the mean; TAA=thoracic aortic aneurysm; Treg=regulatory T-cells

The mean value (± SEM) of blood Treg, expressed as a percentage of total CD4^+^ T-cells, was 1.19±0.09; no statistically significant differences have been observed between the 2 groups of patients. The frequency ratio of CD4^+^CD28^±^CD4^+^/CX3CR1^+^ T-cells *vs*. circulating Treg was significantly lower in the TAA group than in the AVD group (Figure 6).

### CD4^+^ T-cells Phenotype/Function, Aortic Valve Pathology, and Clinical Features

No correlation has been found between all the above-mentioned phenotypic and functional CD4^+^ T-cell features and the patients' ages. Student's t-test and linear regression statistical analysis did not show significant associations between immunological parameters and patients' clinical characteristics, such as the prevalent type of valve pathology, aortic valve mean gradient values, associated risk factors, and current medical therapies. The mean functional ratio of IFN-γ *vs*. IL-17A+IL-21 cytokine-producing CD4^+^ T-cells was significantly higher (*P*=0.0228) in female (2.144±279) than in male patients (1.398±0.147). On the other hand, the Chi-squares statistical analysis did not evidence a significant association between sex differences and the presence of TAA.

## DISCUSSION

Clinical and experimental studies carried out in abdominal aortic aneurysms by histological and immunohistochemical procedures proved that a predominant Th2-mediated immune response, mainly driven by IL-4, IL-5, or IL-10 cytokines, induces severe aneurysm formations^[27]^. On the other hand, a prevalent Th1-mediated immune response, sustained by the infiltration of mononuclear cells releasing IFN-γ and IL-12, has been demonstrated to be responsible for transmural inflammation and external vessel wall dilatation in ascending TAA^[28]^. Nevertheless, independently of their localization, both types of dilating aortic lesions are characterized by common histopathological findings, also including evident modifications of ECM turnover with overexpressed collagen deposition and fibrosis^[2-4]^. As known, the ECM remodeling, with fibroblasts activation and development of fibrosis, occurring in pathological conditions, such as systemic sclerosis, atherosclerosis, parasitic infections, and after the use of silicone mammary implants, is suppressed by a locally polarized Th1 IFN-γ-driven immune response^[5,6]^.

In spite of the histological demonstration of a prevalent Th1-mediated immune response into the wall of dilated and/or aneurysmatic ascending thoracic aortas, so far little is known about the systemic immunological status of these patients. Therefore, in our study, we evaluated several phenotypic and functional features of peripheral blood CD4^+^ T-lymphocytes in patients undergoing a cardiac operation for aortic valve replacement associated, or not, with elective surgery for TAA.

We have found out that the cumulative CD4^+^ T-cell fraction calculated by percentage addition of circulating CD4^+^CD28^-^ T-cells, a subset of cytotoxic T-lymphocytes producing large amount of IFN-γ^[29]^, plus CD4^+^ T-cells carrying the fractalkine receptor CX3CR1 (CD4^+^/CX3CR1^+^), preferentially expressed on Th1 IFN-γ-producing cells^[30]^, is significantly lower in the TAA group than in the AVD group.

A prevalent pro-fibrotic IL-17A/IL-21-driven polarization of blood CD4^+^ T-lymphocytes in the TAA group seems to be demonstrated, in our study, by the significantly lower mean ratio observed between the frequency of CD4^+^ T-lymphocytes producing the anti-fibrotic IFN-γ *vs*. the total fraction of IL-17A+IL-21 pro-fibrotic cytokine-producing CD4^+^ T-cells^[6,31,32]^.

Previous papers demonstrated that human fibrocytes are potent antigen-presenting cells (APC) capable of priming naive T-cells *in situ* ^[33]^. Since the requirement of a more broad T-cell/APC cross-talk via CD40-CD40L interactions for generation of Th17- than for Th1 IFN-γ-mediated inflammatory responses^[34,35]^, our data suggest that the higher extent of fibrotic tissue may orientate aneurysmatic aorta tissue-resident T-cells towards a prevalent production of IL-17A and IL-21, so creating a self-maintaining loop for further fibrocyte priming, collagen production, and pro-fibrotic tissue remodeling.

The close correlation existing between the individual values of the IFN-γ/(IL-17A + IL-21) functional ratio and the CD4^+^CD28^±^CD4^+^/CX3CR1^+^ T-cell fraction indicates that the association of these last phenotypic T-cell subsets is strictly involved in the establishment of a prevalently IFN-γ-oriented blood CD4^+^ T-cell polarization in AVD subjects without TAA.

Moreover, while previous papers showed that Treg and IL-10-producing CD4^+^ T-lymphocytes separately suppress collagen deposition and tissue fibrosis in several chronic inflammatory conditions^[5]^, apparently cooperating with the anti-fibrotic action of IFN-10-producing CD4^+^ T-cells, the significantly lower ratio of total CD4^+^CD28*^±^* CD4^+^/CX3CR1^+^ T-cell *vs*. Treg observed in our TAA patients suggests that a tissue IFN-γ counter-acting effect of Treg, mainly sustained by IL-10 production^[36]^, could be detrimental for this specific pathological condition.

Previous papers have shown that circulating T-cells represent an important repository pool to reveal tissue-resident T-cell functional abnormalities in immune-mediated connective pathologies, such as systemic sclerosis, characterized by a deregulated fibroblast activation leading to fibrosis of internal organs^[37]^. Moreover, it has been demonstrated that the evaluation of balanced dynamic inter-relationships among different phenotypic/functional characteristics of blood CD4^+^ T-cell subsets can identify unique immunological features correlating with the clinical outcome and therapeutic interventions in selected cardiovascular patients^[9]^.

## CONCLUSION

We conclude that there is a presence of an immunological imbalance in the form of fibrocyte activation and Treg differentiation leading to the development of an aortic aneurysm in patients with AVD. On this basis, eventually, a targeted therapeutic model could be developed if it is confirmed in a large number of patients with TAA, including subjects with aortic rupture/dissection.

**Table:** 

Authors' roles & responsibilities	
SS	Work design; concept; experiments; lab work; data acquisition; analysis; interpretation and manuscript writing; final approval of the version to be published
KKT	Work design; concept; experiments; lab work; data acquisition, analysis; interpretation and manuscript writing; final approval of the version to be published
SB	Concept; data analysis; interpretation; supervision; final approval of the version to be published
PG	Lab experiments; advice; review of manuscript and critical appraisal; final approval of the version to be published
EK	Supervision; addition of concept; review of manuscript and critical appraisal; final approval of the version to be published
MS	Funding; logistic; supervision; final approval of the version to be published
AMM	Concept, supervision; final revision and approval of the manuscript; final approval of the version to be published
